# Prenatal Diagnosis of Canine and Feline Twins Using Ultrasound: A Retrospective Study

**DOI:** 10.3390/ani13213309

**Published:** 2023-10-25

**Authors:** Fabiana Pecchia, Stefania Di Giorgio, Alessandra Sfacteria, Salvatore Monti, Cecilia Vullo, Giuseppe Catone, Gabriele Marino

**Affiliations:** 1 Polivet, Via Salaria 1317, 00138 Roma, Italy; pecchia.fabiana@gmail.com; 2 Veterinary Teaching Hospital, Department of Veterinary Sciences, University of Messina, Viale Giovanni Palatucci, 98168 Messina, Italy; sdigiorgio@unime.it (S.D.G.); asfacteria@unime.it (A.S.); salvatore.monti@unime.it (S.M.); cecilia.vullo@unime.it (C.V.); gcatone@unime.it (G.C.)

**Keywords:** dog, cat, twins, ultrasound, dystocia, dysmaturity

## Abstract

**Simple Summary:**

This study focused on the ultrasound diagnosis of twins in canine and feline species. Prenatal diagnosis is becoming increasingly important even in small animals to assess foetal morphology and identify any eventual abnormalities. In canine and feline species, which are typically polytocous species, only monochorionic twins, i.e., sharing at least the same placenta, can be termed, using ultrasound, as twins. In this study, seven cases of monochorionic twins were reported with a prevalence of under 1%. The clinical relevance of such a diagnosis is to prevent complications during pregnancy and, especially, delivery.

**Abstract:**

Prenatal diagnosis comprises a set of investigations, both instrumental and laboratory-based, which aim to monitor the health of the foetus during pregnancy, from the early stages of embryonic development to the moments preceding delivery. A growing interest is emerging for the preterm ultrasound morphological screening of embryos and foetuses, aimed at assessing the integrity and viability of the conceptus, as well as the early diagnosis of anomalies which can cause complications. This study is a retrospective study of the ultrasonographic findings of twins in the authors’ clinical activity from 2016 to 2022. Only seven cases of monochorionic twins were recorded, out of the whole number of evaluations performed on 3120 foetuses, with a prevalence of 0.6% and 0.2% in feline and canine foetuses. All the twins had their own amniotic sac and umbilical cord but presented a single placenta and a single allantoic sac. Unfortunately, the three feline cases were not more recognizable at term. In the four canine cases, three were of opposite sex and then necessarily dizygotic. Twins may have an impact on the success of a pregnancy due to the risk of dystocia, as observed in some of the reported cases. Prenatal ultrasound allows early recognition of twins in dogs and cats.

## 1. Introduction

Prenatal screening or diagnosis consists in the detection of normality or the presence of foetal pathologies of various types which are identified during a gestational period. In the human species, amniocentesis and villocentesis are considered the techniques of choice for prenatal diagnosis; however, their invasiveness carries a minimal risk of miscarriage [1,2]. Among the non-invasive screening procedures, the most common are ultrasonographic evaluations, blood pressure measurements, and the dosage of circulating markers in the pregnant woman in question. Prenatal ultrasound is generally considered a safe method of obtaining diagnostically valid images and allows the detection of foetal abnormalities or an abnormal development of the foetus. Down syndrome, for example, can be suspected based on the evaluation of foetal nuchal translucency and the development of the nose bone [2], as well as abnormal levels of specific proteins in the mother’s blood in the first 15–16 weeks of pregnancy.

In veterinary medicine, prenatal diagnostic techniques are not yet as developed: essentially, they are limited to the evaluation of the mother’s health, the viability and the number of embryos/foetuses, and the estimation of the gestational age. Among imaging techniques, X-ray is still an elective technique in the estimation of litter size and is also able to diagnose foetal death by highlighting abnormal foetal bone displacement and/or gas in or around the foetuses [3,4,5]. Ultrasonography is the diagnostic modality of choice for diagnosing pregnancy, assessing foetal viability, and predicting the day of delivery, as well as for all the morphological evaluations of the foetus, including sexing [6,7,8,9,10]. Increasing attention has been paid to the ultrasound evaluation of the early diagnosis of anomalies. Normal foetal development can be assessed by observing the chronologic relationship between organ development and gestation, which is also useful for determining the date of delivery [10,11,12,13,14,15,16], and embryonic/foetal disorders can be predicted by observing a delay in the time of development of the organs or alterations in their appearance, or by finding a slow rate of foetal growth [17,18,19]. Single reports of foetal abnormalities diagnosed early using ultrasound have been reported, both in canine and feline species, including monochorionic twins [20,21], hydrops fetalis [22,23,24,25,26,27,28], hydrocephalus [29,30], clefts [31], and kidney defects [32].

**Figure 1:**
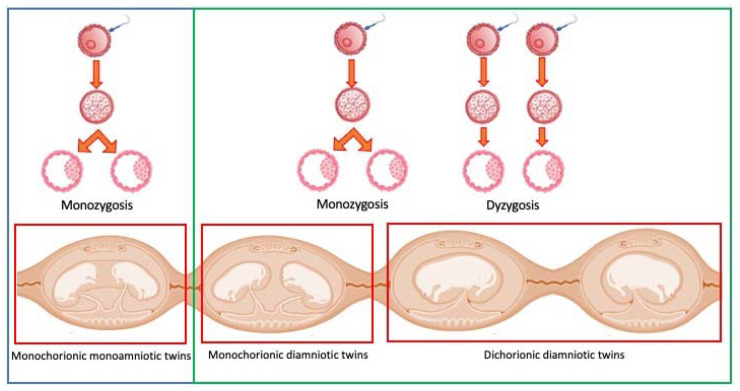
Terminology for twin pregnancies. Twins can be identical (monozygosis) or fraternal (dyzigosis). They can share the same placenta (monochorionic twins) and even the same amniotic sac (monochorionic monoamniotic twins). Monochorionic or dichorionic twins can be identical or not. Monochorionic monoamniotic twins are always identical.

Focusing on twins, a clarification of the terms is necessary. Twin pregnancies are classified according to the type of conception (zygosity), the type of placentation (chorionicity), and the number of amniotic sacs (amnioticity) (Figure 1).

Monozygotic twins (also called identical twins) result from the fertilization of a single egg by a single sperm, with the fertilized egg then splitting into two. Identical twins share the same genome and are always of the same sex. In contrast, dizygotic twins (fraternal) result from the fertilization of two separate eggs with two different sperm during the same pregnancy. Fraternal twins may not be of the same sex. Twins that share a single placenta are called monochorionic twins. A general rule exists in the field of obstetrics according to which monochorionic twins are almost exclusively monozygotic [33]. However, monochorionic dizygotic twins have been reported in humans after assisted reproductive technologies [34] and in dogs [20]. On the other hand, dichorionic dizygotic twins are the norm, and dichorionic monozygotic twins are extremely rare [35]. Twins that share a single amnion are called monoamniotic twins. They are all monozygotic. Monochorionic diamniotic twins are almost always monozygotic, with a few exceptions where the blastocysts have fused [36]. Then, a correct diagnosis requires a genetic assessment and a careful evaluation of the gestational sacs, the latter also in uterus, by means of imaging techniques.

During a wider retrospective study on prenatal diagnoses of foetal abnormalities in dogs and cats, the ultrasonographic findings and follow-up of seven new cases of monochorionic twins were reported.

## 2. Materials and Methods

A retrospective survey of ultrasound cases of foetal abnormalities in dogs and cats was performed in the authors’ affiliation clinics from 2016 to 2022.

The ultrasound examinations were performed with machines (General Electric Logiq E9—GE Medical Systems, Milan, Italy, and General Electric Versana Active—GE Medical Systems, Milan, Italy) equipped with linear and micro-convex transducers, with frequencies between 8 and 18 Mhz. The enrolled patients were owned animals undergoing routine diagnostic investigations during pregnancy. Informed consent was obtained from each owner. No sedation was necessary during the evaluations. A first check was performed at different gestational ages, although an early diagnosis was always suggested. The stage of pregnancy was calculated, in the female dogs, using the day of ovulation, directly detected with ovarian ultrasound and/or indirectly using the values of progesterone from 5 to 8 ng/mL; instead, in the female cats, the stage of pregnancy was calculated using the day of mating. The examinations were ideally carried out at weekly intervals, until the birth, although the owners did not always keep the appointments, extending the interval. The gestational age was confirmed or adjusted by measuring embryo-foetal and extra-foetal biometric parameters according to the stage of gestation [11,12].

For this study, the morphological examination started from day thirty of pregnancy and included the evaluation of each gestational chamber. Special attention was paid to the zonate placenta, evaluated for contour, thickness, echogenicity, vascularization, detachment points from the uterus, and origin of the umbilical cord. The number of embryos/foetuses was checked in relation to the gestational sacs, umbilical cords, and placentae, to detect any resorptions or cases of twins. Only foetuses sharing the same allantoic sac (monochorionic twins) were diagnosed as twins.

The data were collected from four different trained operators (G.M., F.P., G.C., and C.V.). The critical points of the examination were as follows: the missing of a twin; the inability to count and assess all the embryos or foetuses correctly all the time; and the repetition of the examination in the same gestational sac.

The prevalence was calculated as the number of twins on the number of explorable foetuses using ultrasound and as the number of twins on the number of pregnancies separately for the feline and canine species. Fisher’s exact test was used to compare the prevalence of abnormalities between the canine and feline species with 95% of confidence.

## 3. Results

This study reports the findings on 600 ultrasonographic evaluations, on 440 female dogs and 160 female cats of different breeds and ages. A total of 3120 foetuses were morphologically examined (2640 canine and 480 feline). Seven cases of twins were identified, three twins in two female cats and four in four female dogs. There was a prevalence of 0.6% and 0.2% (*p* = 0.2) in the feline and canine foetuses and of 1.9% and 0.9% (*p* = 0.6) considering the mothers. No significant differences between the two species were demonstrated.

**Figure 2:**
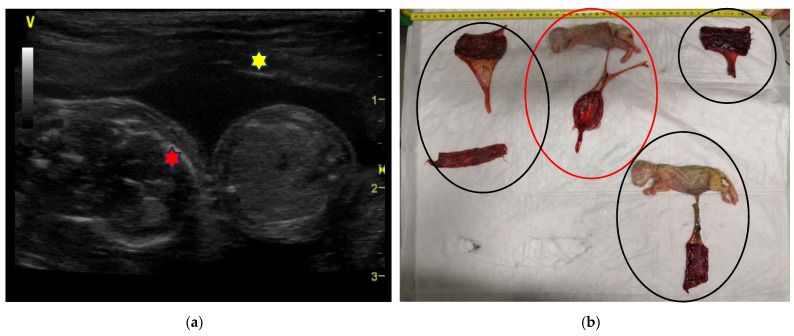
Twins in a Sacred Birman female cat: (**a**) Ultrasound (linear probe 12 MHz) showing two viable foetuses sharing the same gestational sac. In the left, the foetus’ bones of the skull are easily detectable (red star); the right foetus is scanned at the level of the liver; both foetuses share the same placenta (yellow star). The estimated gestational age is 45 days. (**b**) At delivery, a dead kitten (red circle) was connected to its placenta by the umbilical cord, while another umbilical cord connected to the same placenta was truncated. Another dead kitten and three remnants of placenta (black circles) were found. The placental remnants were non easily recognizable; they were probably fragmented; however, at birth, the second couple of twins was not confirmed.

The first two cases were found in a 6-year-old Sacred Birman female cat, weighing 3.6 kg. At the diagnosis of pregnancy performed for the first time 45 days from mating, two couples of monochorionic twins were identified (Figure 2a). In the left uterine horn, two adjacent gestational chambers were observed, each containing two live and viable foetuses; in the other horn, a single foetus was detected normally lodged in its own gestational sac. The measurement of the biparietal diameter (BD) was similar for all the foetuses and allowed the operator to calculate the date of expected delivery (BD = 23.39 + 0.47 days). An elective caesarean section (C-section) was planned, but the cat delivered alone, at home, the night before the date of the surgery. Out of the five kittens born, three were found dead. One of the latter was still connected by the umbilical cord to its placenta, from which a second truncated umbilical cord was seen and recognized it as a twin (Figure 2b).

In the other kittens, the umbilical cord had been severed by the mother. Of the two kittens who had been born alive, only a female kitten was weaned because the other died seven days after birth from neonatal sepsis.

**Figure 3:**
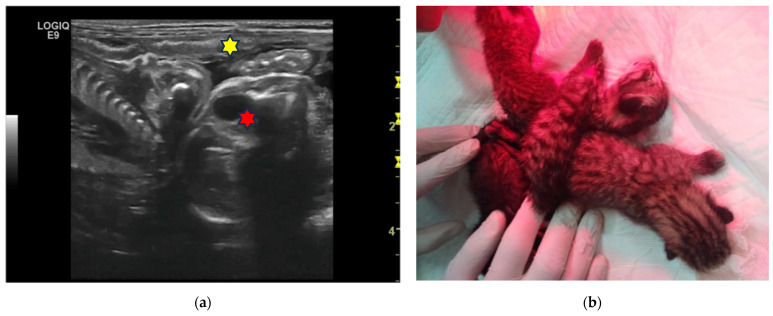
Twins in a European cat: (**a**) An ultrasound (linear probe 12 MHz) scan found two foetuses sharing the same gestational sac (yellow star). They appear very close, tail to tail. The foetal bladder (red star) is visible in the right foetus. The estimated gestation age is 50 days. (**b**) Tight entanglement of the twins at delivery.

In the third case, a 4-year-old European female cat was evaluated 50 days after natural mating. A couple of twin foetuses were found, sharing the same gestational chamber in the left horn, and two other foetuses, each in its own gestational chamber, were found in the other horn (Figure 3a). The foetuses were alive and viable, with normal heart rates, comparable biometric measurements, and without developmental abnormalities. An elective C-section was proposed but refused by the owner. The cat delivered 15 days later, alone and during the night. In the morning, the owner found the four kittens alive and viable with their umbilical cords still attached and tightly twisted together, to the extent that one of the kittens had their hind leg trapped (Figure 3b).

After cutting the cords, unfortunately, the amputation of the limb was necessary. However, the twins were not more recognizable.

**Figure 4:**
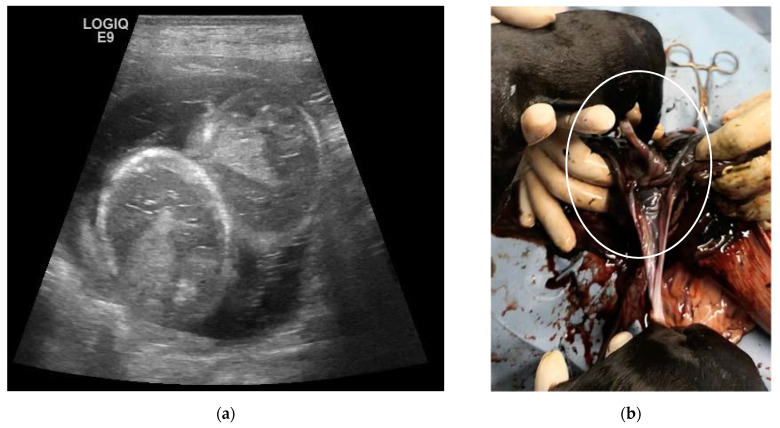
Twins in a Labrador female dog: (**a**) Ultrasound (micro-convex probe 8 MHz) reveals two foetuses sharing the same gestational sac. The foetuses are scanned transversally at lung/liver level. The estimated gestational age is 47 days. (**b**) At C-section, the monochorionic twins are confirmed by the two umbilical cords attached to the same placenta, one of which is partially twisted (circle).

The fourth case concerns a multiparous 5-year-old Labrador Retriever female dog, weighing 39 kg. At the first ultrasonographic evaluation (25 days), ten embryos were evident, two of them within the same gestational chamber. The twins were monitored at weekly intervals and showed normal development, biometry, and viability (Figure 4a).

The umbilical cords were attached to different points in the same placenta, and the foetuses were in opposite presentation in the longitudinal view and maintained this presentation. At the time of the planned C-section, the twins were delivered alive and viable and were found to have two different amniotic sacs. The umbilical cords were connected to the same placenta, and one was partially twisted (Figure 4b). Two other puppies were euthanized for severe cleft palate, malformed limbs, and severe abdominal cleft; another mature puppy was extracted already dead. Unfortunately, the abdominal clefts were not detected during prenatal checks. The twins were of different sex and with a similar size compared to their littermates. The neonatal parameters were in the normal range, but, unfortunately, the male pup died after 7 days of hypoglycaemia and dehydration, but the cause was not clear.

**Figure 5:**
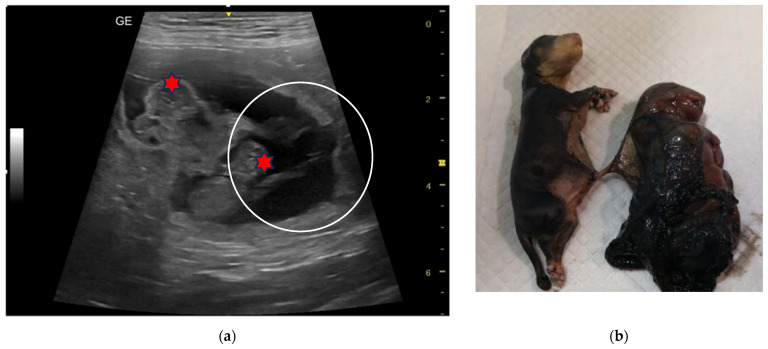
Twins in an Australian Shepherd dog: (**a**) The ultrasound image (micro-convex probe 12 MHz) shows two foetuses, with two distinct heads (red stars), scanned in the same gestational sac, with two umbilical cords (circle). The estimated gestational age at the time of this assessment is 30 days. (**b**) Twins of the opposite sex delivered already dead at the time of C-section. Death probably occurred at different times.

The fifth case is a 3-year-old Australian Shepherd female dog, artificially inseminated with fresh semen and diagnosed as pregnant at 23 days. Twelve embryos were counted, including a couple within the same gestational sac (Figure 5a). As in the previous cases, the embryos showed no signs of distress and had similar anatomical development. After routine monitoring, the date of delivery was scheduled, and a C-section proposed. The owner initially opted for a natural delivery, but the presence of green–brown discharge during the first phases of labour suggested the necessity of an emergency C-section. Ten alive and viable puppies were delivered. The twins were found almost engaged in the birth canal, probably causing an obstructive dystocia. They were of the opposite sex and died at different times, as one was found with the amniotic sac dry and attached to the body (Figure 5b). When compared to their littermates, they appeared slightly reduced in size.

**Figure 6:**
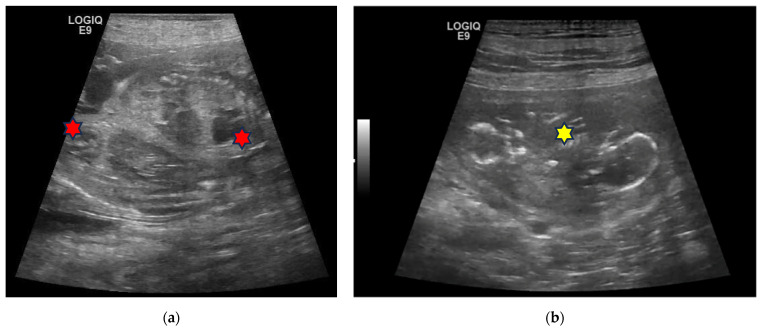
Twins in a Rottweiler dog: (**a**) The ultrasound image (micro-convex probe 8 MHz) shows two foetuses alive and in opposite positions (red stars indicates the hearts), sharing the same gestational sac. The estimated gestational age at the time of this evaluation is 45 days. (**b**) After 10 days, the gestational sac is collapsed; the bones are disarranged (yellow star), and no anechoic fluid is detectable, which are all signs of foetal death.

In the sixth case, during the routine pregnancy monitoring of a 5-year-old Rottweiler female dog, weighing 45 kg, a couple of twins were identified at 45 days of pregnancy, together with two other foetuses. All the foetal parameters were normal and comparable (Figure 6a).

At 55 days, unfortunately, the twins’ sac were collapsed, filled with echoic material; the two foetuses were not mobile and were found without heart beat; the other two foetuses were viable (Figure 6b). A C-section was refused and, effectively, the female dog delivered two alive puppies, but the dead twins were retained. A C-section was necessary. They were both male.

**Figure 7:**
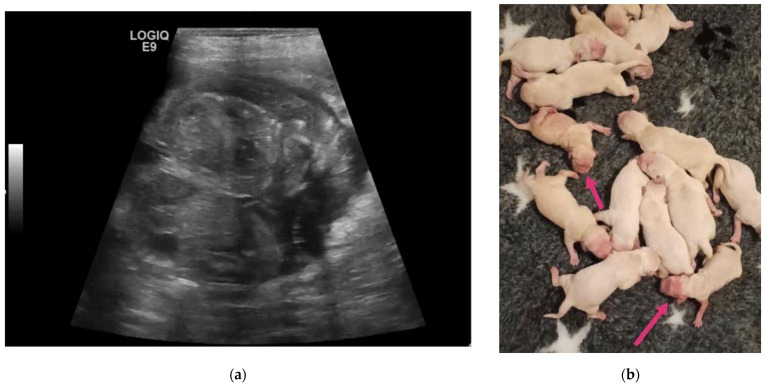
Twins in a Golden Retriever dog: (**a**) The ultrasound image (micro-convex probe 8 MHz) shows two foetuses sharing the same gestational sac at 50 days of gestation. (**b**) The twins at the time of C-section. They appear slightly reduced in size with signs of dysmaturity (arrows).

The last case concerns a 3-year-old Golden Retriever female dog, weighing 32 kg, inseminated via endoscopic transcervical intrauterine artificial insemination with fresh semen, which was diagnosed pregnant at 50 days, with an estimated numerosity by x-ray of 14 foetuses. One couple of twins was found using ultrasound (Figure 7a). An elective C-section was proposed and performed. The twins were delivered alive and were of opposite sex. When compared to their littermates they appeared slightly reduced in size, with areas without hair, and with deep pink discoloured skin on the head and limbs, indicative of a certain degree of dysmaturity (Figure 7b). Subsequently, the twins recovered, and their hair and weight were comparable to their brothers at weaning.

The results are summarized in Table 1.

## 4. Discussion

Nowadays, with the development of high-performance equipment, it is possible to obtain detailed ultrasonographic images and, therefore, better assess foetal morphology, identifying any abnormalities more easily and at an earlier stage. Prenatal diagnosis is thus becoming increasingly important even in small animals, especially when, due to an increased risk of dystocia, an elective C-section is required. The antenatal diagnosis of malformed foetuses or the suspect of dysmaturity could require post-natal care in new-borns. The technique above is applicable in female dogs and cats with pregnancies with a small number of offspring, but it can also be carried out in prolific patients, if cooperative.

The present study focused on the ultrasound diagnosis of twins in canine and feline species. This technique requires more time and experience than a simple pregnancy diagnosis. The operator must pay more attention to the number of embryos/foetuses, umbilical cords, and placentae. In a second step, the examiner should identify amniotic and chorionic sacs. In two cases (4C, 5C), the twins were found in an early period of gestation (23–25 days), when it is easier to count and assess all the embryonic vesicles. The accuracy of the findings is likely to be influenced by the temperament, size, and body condition score of the patient, the number of concepta, their hyperactivity, and the operator’s ability to obtain the correct ultrasonographic scan. The major risk of this procedure is to miss some vesicles or to evaluate the same vesicle twice; this potential bias can interfere with the sensibility of the technique.

Prenatal diagnoses, using ultrasound, of monochorionic twins have been reported in dogs [20,21], but never in cats. However, monochorionic twins have been described macroscopically in dogs at birth [37,38,39]. Instead, different reports describe conjoined twins (monochorionic monoamniotic twins) in cats [40,41,42] and dogs [43,44,45,46,47]. Despite these case reports, this is the first study that attempted to evaluate the prevalence of twins in these species using ultrasonography. The feline cases were described for the first time and showed a higher prevalence than their canine counterparts, although the difference was not significant. There is a risk of underestimating the real prevalence of twins for different reasons: a large average litter size (especially in dogs), a smaller number of C-sections with respect to natural deliveries (especially cats), a difficulty in recognizing twins with spontaneous natural delivery.

Ultrasound was shown to be a useful tool to evaluate twins and monitor their development and viability. The main finding was the presence of two umbilical cords attached to same placenta, with two embryos or foetuses inside the same chorionic sac. All the twins in the study presented their own amniotic sac. There are no reports of monochorionic monoamniotic twins in dogs and cats, excluding conjoined twins. The biometric evaluation of the twins compared to their other siblings showed no differences, but, at term, some twins may present a smaller size, as already reported in the literature [21,39]. In cases 5C and 7C, the twins were lighter and slightly dysmature (7C), but they recovered quickly during lactation (7C). In case 4C, the partial twisting of the umbilical cord occurred in a twin, and, in case 5C and 6C, the asynchronous and synchronous foetal deaths of the twins were observed. Foetal death and anasarca have been already documented in canine twins [20].

A single, shared placenta in monochorionic twins is likely to fail to meet the nutritional needs of both foetuses, and intrauterine growth retardation may occur in one or both twins due to imbalances in the placental circulation, especially near the end of pregnancy, when foetal needs increase. Probably, the reduced surface area of the placenta for the foetus leads to placental insufficiency and to the inability to provide adequate foetal nutrients and oxygen [48].

At delivery, our findings suggest that twins may cause different complications, while normal deliveries of twins have been reported [21,39]. Stillborn or neonatal death is possible [20], as observed in cases 1F, 2F, and 5C. Dystocia was observed in cases 1F, 2F, 5C, and 6C, probably due to the simultaneous presentation of the twins (5C) or due to the presence of one or both dead twins (6C). Both conditions may cause a prolonged delivery or even an obstructive dystocia [49].

This study reports a case (3F) of entangled umbilical cords, involving all the littermates among which a couple of twins was present. This neonatal condition has been anecdotally reported in a Persian cat [50], and the long hair of the mother was considered a predisposing factor. It is here hypothesized that the presence in twins of two umbilical cords connected to the same placenta may easily, at birth, entangle one extremity of another kitten. Moreover, the breed (European) presented short hair.

In consideration of a review of the literature and through the analysis of these new cases, an elective C-section should be strongly considered when a condition of twins is prenatally diagnosed.

Finally, this study did not include a genetic analysis to investigate the zygosity. Monochorionic diamniotic dizygotic twins have been demonstrated dogs [20,39], while there is only one report of monochorionic diamniotic monozygotic twins [38]. In the reported feline cases, the twins were not more recognizable at delivery, which occurred during the night and without assistance; while, in the canine cases, three out of four were of the opposite sex and then certainly dizygotic. In monochorionic twins and, at least, in dogs, dizygosis seems more frequent than monozygosis. In humans, most monochorionic twins are monozygotic; dizygosis has been documented and considered an extremely rare condition [34]. The occurrence of monozygotic dichorionic diamniotic twins is not excluded, although it has never been documented in these species. This extremely rare condition requires genetic testing on all the littermates and has poor or no clinical relevance in a polytocous animal species.

## 5. Conclusions

Prenatal diagnosis using ultrasound is possible and very useful for monitoring the viability of foetuses and twinning, which, although it is a rare condition in dogs and cats, has an impact on the success of a pregnancy. An early diagnosis of pregnancy is always suggested to better evaluate the number of concepta and eventual disorders. More ultrasound evaluations during pregnancy and an elective C- section are strongly suggested when twinning has been detected. Unfortunately, close monitoring is not always possible, like in this case series, due to different limitations, generally due to the owner.

## Figures and Tables

**Table 1:** Cases of the feline and canine twins presented in this study. F: feline; C: canine.

N	Signalment	Day of Pregnancy	Littermate Numerosity	Ultrasound Evaluation	Term of Pregnancy	Follow-Up
1F 2F	6-year-old Birman female cat	45 and 52	5	Two pairs of monochorionic twins, morphologically comparable to the other foetus.	Natural delivery without assistance, three kitten stillborn.	One kitten dead for sepsis after 7 days.
3F	4-year-old European female cat	50	4	Monochorionic twins, morphologically comparable to the other foetuses.	Natural delivery without assistance, entangled neonates’ umbilical cords.	
4C	5-year-old Labrador Retriever female dog	25, 32, 39, 46 and 53	10	Monochorionic twins, morphologically comparable to the other embryos/foetuses.	C-section. Twins of different sex, alive, with partial twisting of the umbilical cord. One stillborn, two puppies euthanatized for malformations.	Death of the male twin after 7 days of hypoglycaemia and dehydration.
5C	3-year-old Australian Shepherd female dog	23, 30, 37, 44 and 51	12	Monochorionic twins, morphologically comparable to the other embryos/foetuses.	Natural delivery, dystocia, C-section. Ten puppies alive and two dead twins of the opposite sex.	
6C	5-year-old Rottweiler female dog	45 and 55	4	Monochorionic twins, morphologically comparable to the other foetuses; foetal death of the twins.	Natural delivery of two puppies, C-section, and extraction of the twins.	
7C	3-year-old Golden Retriever female dog	50	14	Monochorionic twins, morphologically comparable to the other foetuses.	C-section. Twins alive and of opposite sex, slightly dysmature.	

## Data Availability

Data available on request due to privacy or ethical restrictions.
